# Effects of propofol and etomidate anesthesia on cardiovascular miRNA expression: the different profiles?

**DOI:** 10.1186/s12871-018-0610-9

**Published:** 2018-10-24

**Authors:** Youxiu Yao, Ning Yang, Dengyang Han, Cheng Ni, Changyi Wu, Xiangyang Guo

**Affiliations:** Department of Anesthesiology, Peking University Third Hospital, 49 North Garden Road, Haidian District, Beijing, 100191 People’s Republic of China

**Keywords:** Anesthesia, Propofol, Etomidate, Cardiovascular, miRNA

## Abstract

**Background:**

The effects of the intravenous anesthetics propofol and etomidate on circulation are significantly different; however, their differing effects on miRNA expression in the cardiovascular system are not clearly understood. The purpose of this study is to investigate the effects of these two anesthetics on miRNA expression profiles in the heart and blood vessels.

**Methods:**

Rats were randomly divided into a propofol group and an etomidate group. Spontaneous breathing was maintained throughout the anesthesia process and the rats’ oxygen supply was ensured. Heart and thoracic aorta tissue was harvested 3 h after induction. The expression profiles of cardiovascular miRNAs were detected by microarray 4.0 analysis. Twelve representative miRNAs were selected for qRT-PCR validation, and their target genes were predicted using bioinformatics methods.

**Results:**

Microarray analysis showed 16 differentially expressed miRNAs in heart tissue from the propofol group compared with the etomidate group (10 up-regulated and 6 down-regulated), while in the blood vessels there were 25 altered miRNAs (10 up-regulated, 15 down-regulated). After verifying 12 representative miRNAs via qRT-PCR, the results showed no significant difference in the expression of miRNAs in the heart tissue, but a significant difference in the expression of 5 miRNAs in vessel tissue between the two groups. Bioinformatics analysis predicts that the target genes of the 5 differentially expressed miRNAs are associated with chemical synapse signaling pathways.

**Conclusions:**

Propofol and etomidate have different effects on the expression of cardiovascular miRNAs, and further research is needed to elucidate the functional consequences of these differentially expressed miRNAs.

**Electronic supplementary material:**

The online version of this article (10.1186/s12871-018-0610-9) contains supplementary material, which is available to authorized users.

## Background

Propofol and etomidate are two different intravenous anesthetics, which have different effects on circulatory inhibition and cardiovascular function [1–3]. These differences are especially important in elderly or critically ill patients, or patients with cardiovascular diseases [1, 4], and the mechanisms of these different effects on circulation have attracted the attention of a great many scholars [5–7]. Hypotension induced by propofol anesthesia may be associated with inhibition of the sympathetic nerve baroreceptor reflex, while etomidate permits retention of this autonomic regulation [5]. High concentration anesthetics can inhibit human myocardium contraction [6], in a dose dependent manner. In addition, some scholars have studied the negative inotropic and vasodilatation effects of propofol and etomidate, which may be related to the inhibition of L type calcium [8] and potassium channels [8, 9], particularly affecting calcium channels and calcium ion flow [8, 10].

MiRNAs are small, noncoding RNAs 20–22 nucleotides in length that can induce mRNA degradation or inhibit post transcriptional translation, regulating the expression of related proteins, which are regarded as post transcriptional regulatory factors that modulate cell proliferation, differentiation, stress and inflammation [11, 12]. Our previous studies have shown that cardiac miRNAs are released into the circulation after myocardial injury, and may be used as biomarkers of perioperative myocardial injury [13]. MiRNAs in tissues and the circulatory system can also affect cardiovascular function, and can be considered as target points for new drugs [14–18]. Some scholars have reported that miRNA plays an important role in circulatory regulation [18] and in myocardial β receptor signaling pathways and L-calcium channels [19–23].

We hypothesized that miRNA had an effect on cardiovascular function during the peri-anesthesia period, while the intravenous anesthetics propofol and etomidate exert different effects on cardiovascular miRNA expression at equivalent induction doses, hence our decision to conduct microarray and qRT-PCR analysis. The differences in the cardiovascular miRNA expression profiles of these two intravenous anesthetics were detected in order to find the related small RNA molecules; in this way, our study contributes to research on the mechanisms of circulation-inhibiting anesthetics.

## Methods

### Animal model preparation and specimen acquisition

The experiment was approved by the Animal Ethics Committee of Peking University Health Science Center (approval number LA2015172). Six ten-week-old male Sprague Dawley (SD) rats (Beijing Vital River Laboratory Animal Technology Supply) weighing 400 ± 10 g were randomly divided into two groups: a propofol group (group P) and an etomidate group (group E), each group containing 3 rats. The rats in each group were intraperitoneally injected with propofol 100 mg/kg or etomidate 10 mg/kg for basal anesthesia. After 15 min, the rats were in a state of sleep.

The rats were fixed on the plate, and the femoral vessels were dissected locally. Right lateral femoral vein puncture and catheterization were performed under direct vision, and normal saline was infused at 1 ml/h. Invasive arterial pressure monitoring was performed on the left femoral artery. Arterial blood pressure, anal temperature, heart rate, and oxygen saturation were monitored throughout the experiment. Once the animal model and vital signs were stable, a small animal echocardiography was performed, after which an induction dose of 0.1 ml was injected twice via vein catheter. The intravenous induction dose was 2.5 mg/kg propofol (Fresenius Kabi Austria GmbH) in the P group, and 0.25 mg/kg etomidate (Nhwa Pharma. Corporation) in the E group.

An echocardiography was performed immediately after the two induction doses. During the experiment, oxygenation was guaranteed by use of an improvised oropharynx airway inserted into the rats, and a purpose-made mask, which was used to supply pure oxygen. The rats maintained autonomous respiration under anesthesia and the oxygen saturation remained above 90%, with the body temperature holding at 37 °C with the aid of a heat light.

All rats were terminated 3 h after the two induction dose injections. Median sternotomies were immediately performed to obtain the heart and thoracic aorta, which were washed twice with phosphate buffer. Samples were placed in labeled cryopreservation tubes, and stored in a liquid nitrogen tank, the whole process being controlled to 3 min. After 1 day, snap frozen samples were used to extract RNA for subsequent experiments.

### RNA extraction and quality control

Complete RNA samples isolated from heart and vessel samples of each group were extracted using a mirVana™ RNA Isolation Kit (Applied Biosystems). Total RNA was quantified using a NanoDrop ND-2000 (Thermo Scientific) spectrophotometer, and RNA integrity was assessed by agarose gel electrophoresis. A260/A280 nm ratios of 1.7–2.1 indicated that RNA quality was suitable for quantitative analysis and for use in miRNA array and qRT-PCR experiments.

### Affymetrix miRNA 4.0 Array hybridization test

To elucidate the influence of propofol and etomidate on miRNA expression in the cardiovascular system, miRNA expression levels in each group were assessed using an Affymetrix Multispecies miRNA 4.0 Array (Affymetrix, Santa Clara, CA, USA). This array fully covers all 557 known unique rodent miRNAs according to miRBase version 20.0.

First, poly (A) tailing was performed on all the extracted RNA; this was then followed by FlashTag™ Biotin HSR ligation, hybridization, washing and staining. Finally, the arrays were scanned using an Affymetrix Scanner 3000 (Affymetrix 7G) to obtain the original image.

Data analysis was performed on the 12 samples. Command Console software (version 4.0, Affymetrix) was used to analyze the original array images to obtain the raw data. RMA (Robust Multi-chip Analysis) was then applied to the data using TAC (Transcriptome Analysis Console) software, involving 3 steps, namely, background adjustment, quantile normalization, and summarization [24]. In order to identify the differential expression profiles within the different variants, a one-way ANOVA was performed using Transcriptome Analysis Console (4.0) software. The miRNAs were screened according to the threshold, and the miRNA expressions were unsupervised hierarchically clustered and plotted onto volcanic maps. A heat map is normally used to visually represent high dimensional data, with different intensities of expression values represented by different colors within the two-dimensional images. It is usually used in gene expression analysis to indicate the level of gene expression in different samples. The heat maps for this study were constructed to graphically display the results of hierarchical clustering using TIGR MeV (Multiexperiment Viewer) software [25].

### Standard qRT-PCR of representative miRNAs

To verify the accuracy of differentially expressed miRNAs regulated by propofol and etomidate in heart and vessels identified by the microarray data, we performed SYBR green qRT-PCR test analysis on 12 representative miRNAs (rno-mir-129-5p, rno-mir-93-5p, rno-miR-3584-5p, rno-miR-377-3p, rno-miR-425-5p, rno-miR-133b-3p, rno-miR-140-3p, rno-miR-320-3p, rno-miR-24-3p, rno-miR-376c-3p, rno-miR-103-3p, and rno-miR-107-3p). U6 was used as an internal reference control for normalization. Real-time PCR reactions were run in triplicate in 96-well plates on an ABI 7900HT Fast Real-Time PCR System as described elsewhere [26]. The relative quantification Ct method was used to compare the relative amounts of all miRNAs between different groups.

The quantification data was expressed as the threshold cycle (Ct) value. Ct represents the fractional cycle number in which the sample fluorescence signal passes a threshold value above the baseline. ΔCt refers to the difference between the experimental samples and the control, and ΔΔCt represents the difference between the two groups, as calculated by the formula: ΔΔCt = ΔCt of P group − ΔCt of E group. 2^−ΔΔCt^ was used to give the fold change.

### MiRNA target gene prediction and signal pathway analysis

The gene targets for the validated differentially expressed miRNAs from heart and vessels of both P and E groups were located using the MiRTarBase Database version 6.0 [27]. GO (gene ontology) classification and KEGG (Kyoto encyclopedia of genes and genomes) pathway enrichment analysis [28] were performed using DAVID (Database for annotation, visualization, and integrated discovery) software in order to fully interpret the biological function or pathway associated with the major differences in the miRNAs [29].

### Statistical analysis

All results from the data analysis are shown as mean ± standard deviation (SD). An ANOVA test, followed by Tukey’s post hoc test was used to compare the quantitative data between groups. A *p* value of less than 0.05 (two-tailed) was considered to indicate a statistically significant difference. Graph displays were performed using GraphPad Prism Software version 5.0.

## Results

### Physiological data

All rats survived until terminated, and all animal data were used. In this experiment, we administered etomidate (0.25 mg/kg) and propofol (2.5 mg/kg) to simulate the clinical induction dose, since these dosages are clinically equivalent, and there is a difference in circulatory inhibition. We observed that the P group showed significant decreases in blood pressure and heart rate after induction compared with the E group, with no obvious difference in other physiological data, i.e. blood oxygen saturation and body temperature (see Additional file 1: Table S1). Due to our supporting measures, neither hypoxia nor hypothermia occurred in either group.

### Affymetrix miRNA expression profiles

Affymetrix array analysis showed that there were 16 differentially expressed miRNAs in the heart tissue of the P group, including 6 up-regulated and 10 down-regulated, compared with E group, while there were 25 differences in miRNA expression in the vessel tissue of the P vs. E groups; 10 miRNAs were up-regulated and 15 down-regulated (Table 1). The original data from Affymetrix is shown in Additional file 2: Table S2 and the accompanying boxplots.

**Table 1:** Differential changes of miRNA Expression Using Affymetrix Arrays

Heart (Propfol vs.Etomidate)			Vessel (Propfol vs.Etomidate)		
Assay	FC	*P* Value	Assay	FC	*P* Value
rno-miR-105	1.53	0.0028	rno-miR-743a	1.42	0.0031
rno-miR-3573	−1.51	0.0035	rno-miR-24-3p	−1.87	0.0037
rno-miR-129-5p	−1.6	0.012	rno-miR-344a-2	−1.57	0.0041
rno-miR-93-5p	1.73	0.012	rno-miR-3065	1.51	0.0085
rno-miR-3584-5p	−2.27	0.0168	rno-miR-500-3p	1.53	0.0094
rno-miR-203b	1.66	0.0221	rno-miR-184	−1.52	0.0106
rno-miR-139	1.72	0.0248	rno-miR-324-3p	1.5	0.0106
rno-miR-3072	−1.56	0.0251	rno-miR-107-3p	−1.88	0.0124
rno-miR-675-5p	1.54	0.0287	rno-miR-320-3p	−2.42	0.0139
rno-miR-6216	−1.94	0.0297	rno-miR-124-3	−1.52	0.0161
rno-miR-544	1.53	0.0299	rno-miR-124-1	−1.52	0.0161
rno-miR-299b	1.56	0.0311	rno-miR-124-2	−1.52	0.0161
rno-miR-425-5p	−2.31	0.042	rno-miR-3566	−1.43	0.0172
rno-miR-377-3p	1.58	0.0422	rno-miR-221-5p	1.44	0.0172
rno-miR-344a-1	1.54	0.0463	rno-miR-582-3p	1.42	0.0191
rno-miR-3575	1.51	0.0496	rno-miR-6319	−1.47	0.0225
			rno-miR-185-5p	−1.81	0.0226
			rno-miR-1224	−1.54	0.0307
			rno-miR-103-3p	−1.54	0.0312
			rno-miR-133b-3p	2.98	0.0328
			rno-miR-140-3p	−4.08	0.0329
			rno-miR-382-5p	1.41	0.035
			rno-miR-376c-3p	1.43	0.0388
			rno-miR-3541	1.53	0.0389
			rno-miR-6324	−1.43	0.0443

miRNAs were significantly upregulated or downregulated by the propofol anesthesia group compared with the etomidate group by means of Affymetrix arrays, respectively. Values are the mean fold change in each group

*FC* fold change, *miR* microRNA, *rno* Rattus norvegicus

**Fig. 1:**
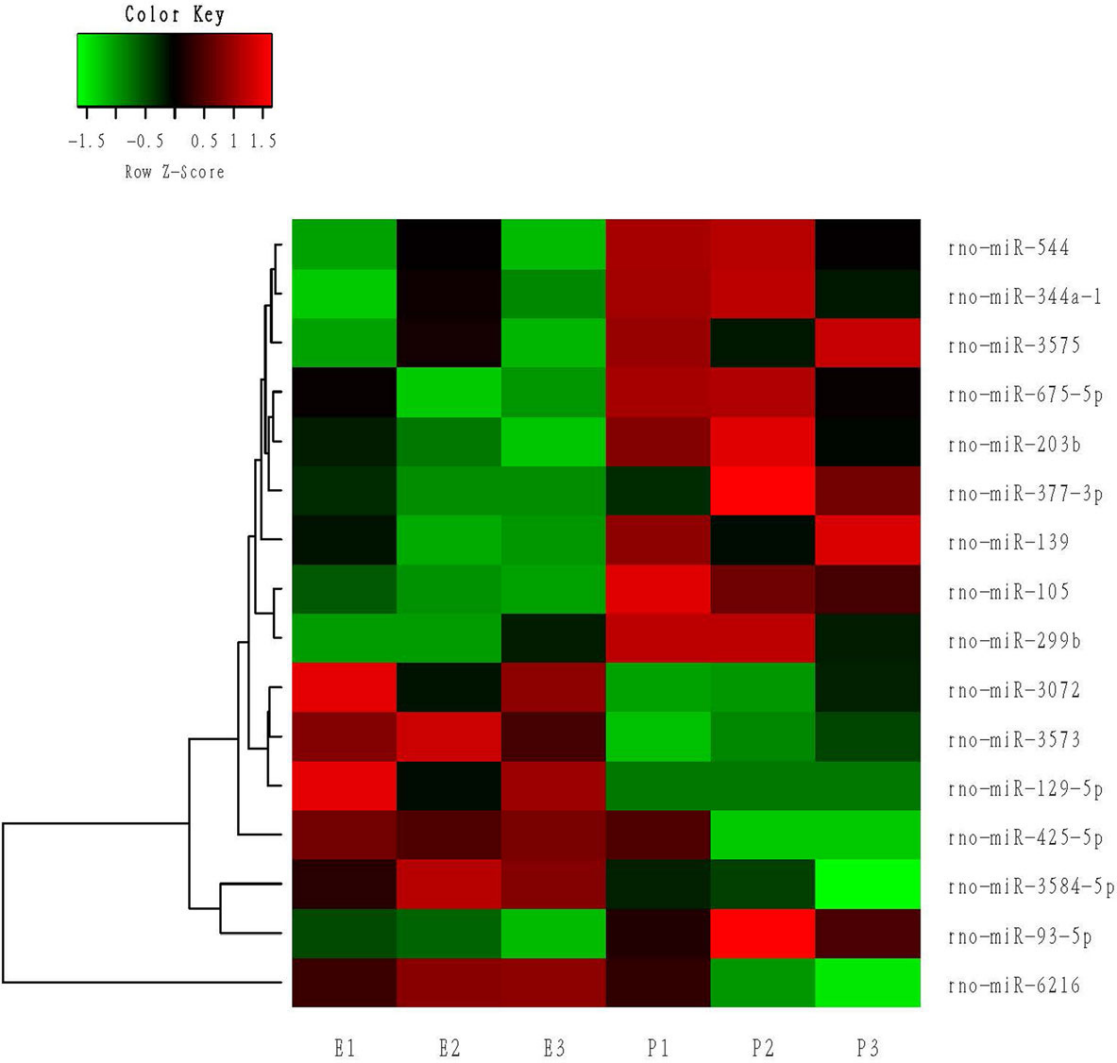
Unsupervised hierarchical cluster analysis of 16 microRNAs (miR) differentially expressed in heart tissues in response to anesthetic agents based on their relative expression levels. Columns correspond to samples and are labeled to indicate whether they represent samples from the etomidate group (E) or the propofol group (P). Each row corresponds to an individual miR. The miR names are given on the right, while the dendrogram for miR clustering is displayed on the left. The color key displays miR expression variance: red indicates a higher gene expression, green indicates lower expression, and black indicates the median value. The samples are divided into two groups: the etomidate group (E1, E2, E3), and the propofol group (P1, P2, P3)

**Fig. 2:**
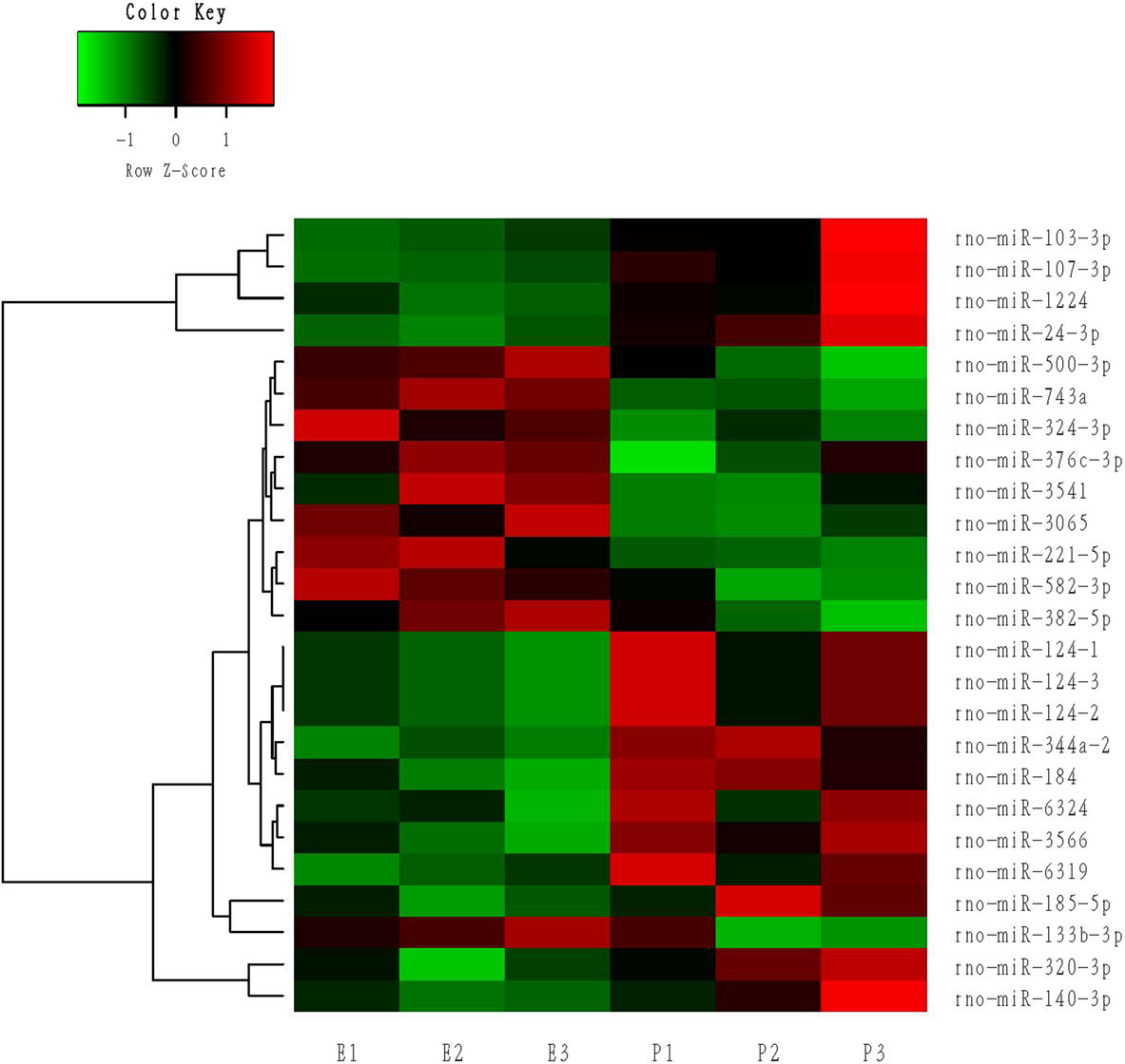
Unsupervised hierarchical cluster analysis of 25 microRNAs (miR) differentially expressed in vessels in response to anesthetics based on their relative expression levels. Columns correspond to samples and are labeled to indicate whether they represent samples from the etomidate group (E) or the propofol group (P). Each row corresponds to an individual miR. The miR names are given on the right, while the dendrogram for miR clustering is displayed on the left. The color key displays miR expression variance: red indicates a higher gene expression, green indicates lower expression, and black indicates the median value. The samples are divided into two groups: the etomidate group (E1, E2, E3), and the propofol group (P1, P2, P3)

Next, heat maps were generated to display the hierarchical clustering analyses of samples and the markedly changed expression of the 16 and 25 miRNAs, as shown in Figs. 1 and 2 respectively. After clustering the columns (samples) and rows (miRNAs), the heat map can display the clustering results of samples and miRNAs simultaneously in a single graph. The intensity of changes in miRNA expression is indicated by the use of different colors. These differentially expressed miRNAs clearly distinguish the P group samples from those of the E group (Figs. 1, 2).

### Validation of Affymetrix microarray results by real-time PCR

**Fig. 3:**
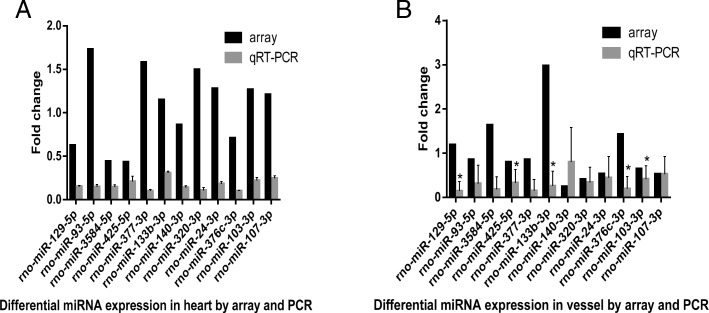
Differential miRNA expressions validated by Affymetrix array and qRT-PCR, respectively. **a** indicates no significant difference between two groups validated by qRT-PCR (*p* > 0.05); **b** indicates 5 miRNAs (rno-miR-129-5p, rno-miR-133b-3p, rno-miR-376c-3p, rno-miR-103-3p, miR-425-5p) significantly decreased in the propofol group vs. the etomidate group following validation by qRT-PCR (* indicates *p* < 0.05)

To verify the results of our Affymetrix array analysis, we carried out qRT-PCR experiments. 12 representative miRNAs were tested from each of the heart and vessel samples. For the heart tissue, 5 miRNAs had been screened as differentially expressed by the array analysis, while 7 had shown no significant difference; in the vessel tissue, 7 had been screened as differentially expressed, while 5 had no significant difference. The original qRT-PCR data is provided in (Additional file 3: Table S3, Additional file 4: dissociation curve). Following PCR verification, there was found to be no significant difference between the two groups in the 12 representative miRNAs extracted from heart tissue (*p* > 0.05, Fig. 3a). For the vessel tissue, 5 of the 12 miRNAs (rno-miR-129-5p, rno-miR-133b-3p, rno-miR-376c-3p, rno-miR-103-3p, and miR-425-5p) were found to be significantly different between the two groups (*p* < 0.05). The array analysis had suggested a difference in the case of 3 of these 5, while 2 had not appeared differentiated (Fig. 3b).

### Gene ontology and KEGG pathway analysis of PCR-verified differentially expressed miRNAs

We studied the functions of the validated miRNA target genes, which were divided into several biological processes, including membrane depolarization, regulation of membrane potential, regulation of postsynaptic membrane potential, membrane depolarization during action potential, and molecular functions including gated channel activity (Additional file 5: Figure S1).

**Fig. 4:**
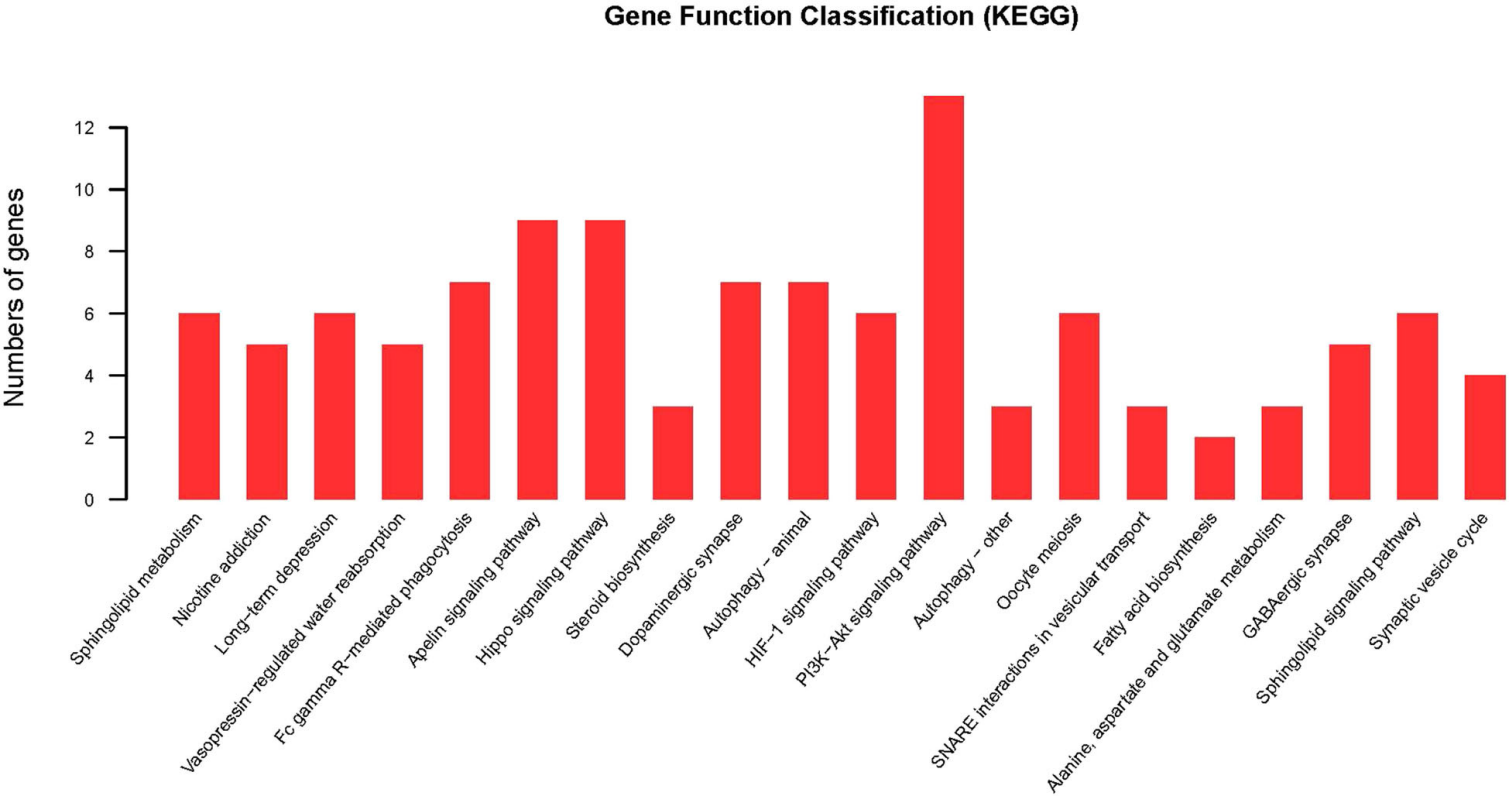
Barplot of KEGG pathway enrichment

**Fig. 5:**
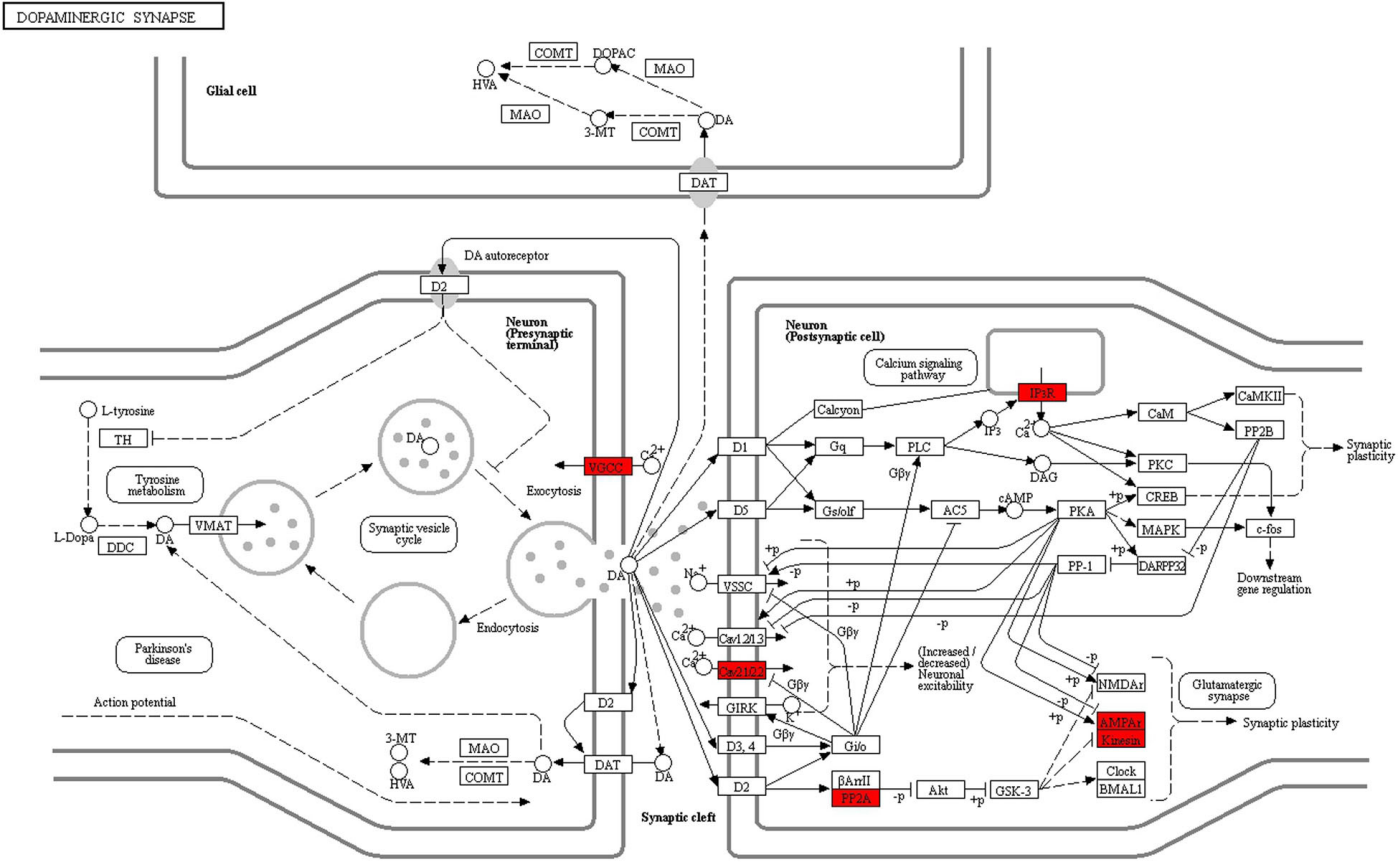
GABAergic synapse signaling pathway, with the enriched target genes highlighted in red. The pathway plot was generated by KEGG (Kyoto encyclopedia of genes and genomes, https://www.kegg.jp/pathway/rno04727). Our thanks to Kanehisa Laboratories for permission to use and adapt it

**Fig. 6:**
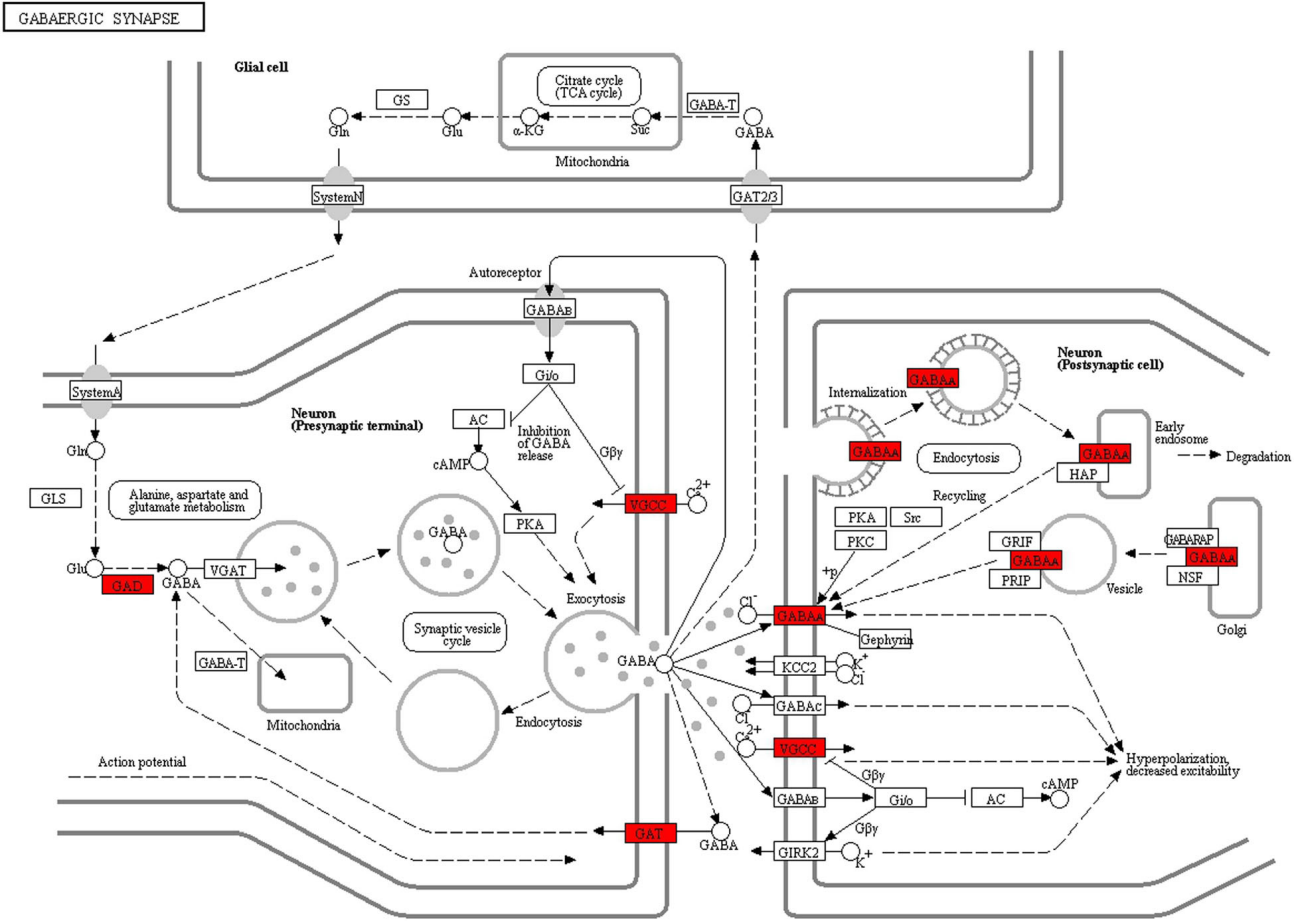
Dopaminergic synapse signaling pathway, with the enriched target genes highlighted in red. The pathway plot was generated by KEGG (Kyoto encyclopedia of genes and genomes, https://www.kegg.jp/pathway/rno04728). Our thanks to Kanehisa Laboratories for permission to use and adapt it

Using DAVID, we found that the miRNA target genes were clustered into pathways, including the PI3K-Akt signaling pathway, HIF-1 signaling pathway, mTOR signaling pathway, dopaminergic synapse, GABAergic synapse, and synaptic vesicle cycle (Figs. 4, 5, 6, and Additional file 6: Table S4).

## Discussion

The molecular mechanism by which anesthetics affect the cardiovascular system is not yet clear. MiRNAs are now recognized as key regulators of cell proliferation, differentiation, metabolism, inflammation, and stress, and miRNA therapy has become a new target treatment for heart failure [18, 30]. In view of the cardiovascular effects of anesthetics, the association between anesthesia and cardiovascular miRNAs deserves more attention. Our study reveals for the first time that two anesthetic agents with different cardiovascular effects produce a difference in the expression profiles of cardiovascular miRNAs. After careful analysis of the miRNA expression profiles in heart and vessel tissue using microarray analysis, we verified the difference using real time PCR. The results showed that two intravenous anesthetics resulted in different miRNA expression profiles in vessel tissue, but no significant difference in cardiac tissue. This confirms that, at least at the miRNA level the different circulatory inhibition effects of propofol and etomidate are not due to the heart, but to the differing effects of the anesthetics on the blood vessels.

The 5 statistically significant differentially expressed miRNAs were found to be down-regulated after propofol vs. etomidate anesthesia. Although the fold change in miRNA expression was not great, we think it is still an important discovery that these small molecular RNAs may play a key role, and we feel this discovery is a preliminary contribution to future mechanism research on the effects of anesthetics on circulatory control. A single miRNA may regulate multiple target genes, and multiple miRNAs can also regulate the same target gene, so the network is complex [11, 13]. We analyzed the target genes of the differentially expressed miRNAs rno-miR-129-5p, rno-miR-133b-3p, rno-miR-376c-3p, rno-miR-103-3p, and miR-425-5p, and considered which target gene(s) might play a role in the signal transduction regulating circulation.

In light of our identification of calcium channels (Cacna1b, Cacnb2, Cacna2d1, Cacng2) as target genes for the above-mentioned down-regulated differentially expressed miRNAs (rno-miR-129-5p, rno-miR-133b-3p, rno-miR-376c-3p, and rno-miR-103-3p), we speculated that the different circulatory inhibition effects of propofol and etomidate are connected to expression changes in miRNA-mediated calcium channels which modulate the conduction of electrical signals or excitation-contraction coupling of the myocardium and vascular smooth muscle. Thus the role of small molecular RNA in the mechanism of circulatory inhibition by anesthetics is intriguing and deserves further research. Our review of the literature did not reveal the mechanism for the cardiovascular effects of miRNA changes caused by anesthetics, although there are some miRNA research reports on heart failure. MiR-24 mediated inhibition of JP2 expression provides a novel molecular mechanism for excitation-contraction coupling in cardiomyocytes [31], and in vivo suppression of miR-24 can prevent decreases in Ca^2+^ transients and L-type Ca^2+^ channel-ryanodine receptor signaling fidelity/efficiency [32]. Wahlquist et al. ascertain that miR-25 modulates SERCA2a, affecting calcium dynamics and impairing cardiac function [18]. Although some studies have reported that miRNAs regulate Ca^2+^ dependent intracellular signals, these avenues of miRNA study are largely unexplored and represent key areas of future research [33]. Based on our preliminary results, we predict that it may be a compensatory callback mechanism producing these miRNA changes under propofol vs. etomidate anesthesia, which may modulate calcium channel genes. The details of this mechanism will constitute our future research direction.

The corresponding target genes were analyzed by GO, with the molecular function involved gated channel activity forming the main focus of our interest. KEGG analysis was also performed, despite the large number of target genes involved in the PI3K-Akt signaling pathway (Fig. 4), our anesthetic research interests focused on chemical synapse, such as the dopaminergic and GABAergic synapses mentioned above, which were the focus points of our further anesthetic research (Figs. 5, 6). The enriched target genes of miRNA identified in this study are shown in red; these include the VGCC and GABA_A_ receptors in the signaling pathway, which may play an important role in the mechanism of propofol circulatory regulation, affecting the electrical signal conduction of cardiovascular nerve cells. Accordingly, we infer that miRNA regulates the effects of VGCC and GABA_A_ receptors on chemical synapses. Our study has shown that propofol and etomidate have different effects on the body, reflected in these signaling pathways, and suggests that propofol may regulate the ion channels through miRNAs, as miRNAs are the key molecules regulating cardiovascular signaling pathways [34]. This further illustrates the complexity and marvel of this regulatory network. We have suggested that the obviously different phenotypes of propofol and etomidate circulatory inhibition may potentially be explained by differences in the internal molecular mechanisms or the subsequent effects on the regulation of gene expression.

We acknowledge that arrays only play a screening role in the detection of miRNAs, and may produce a certain level of false positives, and so at this stage results should be based on quantitative fluorescence PCR. Although all miRNAs screened by array appeared to be down-regulated in PCR, and though the results of PCR and array analysis were not completely consistent, our statistical analysis of *p* values found only 5 miRNAs to be statistically significant (*p* < 0.05). Nevertheless, array screening is still important, as it determines the miRNAs to be verified and identifies key gene alterations. In sum, this study found small molecular miRNAs differentially expressed in the two groups, and showed that these miRNAs’ targets involved calcium channel genes, discoveries that may be of value to subsequent signaling pathway research.

Limitations of our study: 1) Biological reproducibility might be better if the sample size was larger. 2) In this study, samples were taken 3 h after administration of the anesthetics. However, this time point may not represent the peak time of alteration in miRNA expression profiles in tissues: it is possible that miRNAs display more obvious changes at some later time, or may reach peak change earlier and have largely returned to their original states after 3 h. 3) Propofol can lead to circulatory inhibition, reflected in a decrease in blood pressure. This hemodynamic change does not persist, however, and blood pressure will have already returned to baseline levels by *t* = 3 h. It is unclear whether miRNA changes are caused by a decrease in blood pressure, or if propofol affects blood pressure through miRNA alterations. The relationship between the two warrants further research. If other vasoactive drugs such as antihypertensives are used to provide a controlled observation of the effect on the expression of miRNAs, concerns are that the antihypertensive drugs themselves may also affect the expression of microRNAs, which would be difficult to identify. It is difficult to study the relationship between hemodynamics and microRNAs in vivo while avoiding the effects of drugs. Whether changes in microRNAs are caused by decreased blood pressure or by the effects of drugs, the whole process is accompanied by anesthetics. In the presence of anesthetic drugs, which of miRNAs and hemodynamics will change first? Specifically, anesthetics may cause hemodynamic changes through microRNAs, or, more likely, transient hemodynamic changes caused by anesthetics induce compensatory changes in microRNA expression, or both. The specific deeper mechanism is a more complex subject.

However, this study provides the first insight into changes in cardiovascular miRNA expression induced by anesthesia, and makes a preliminary contribution to subsequent mechanism investigations.

## Conclusions

The results of our study reveal that propofol and etomidate anesthesia induce different miRNA expression profiles in blood vessels, but not in cardiac tissue. It is postulated that these differences in gene expression play a key role in regulating circulation. Further studies using miRNA analogues, antagonists, or transgenic animal models are needed to experimentally determine the serial function of each miRNA.

## Additional files

Additional file 1: **Table S1.** Physiological Data for the Propofol and Etomidate Anesthesia Groups. (DOCX 18 kb)

Additional file 2: **Table S2.** Affymetrix original data. Boxplot (vascular). Boxplot (heart). (ZIP 792 kb)

Additional file 3: **Table S3.** qRT-PCR data. (XLSX 40 kb)

Additional file 4: Dissociation curve for miRNA rno-miR-24-3p. Dissociation curve for miRNA rno-mir-93-5p. Dissociation curve for miRNA rno-miR-103-3p. Dissociation curve for miRNA rno-miR-107-3p. Dissociation curve for miRNA rno-mir-129-5p. Dissociation curve for miRNA rno-miR-133-3p. Dissociation curve for miRNA rno-miR-140-3p. Dissociation curve for miRNA rno-miR-320-3p. Dissociation curve for miRNA rno-miR-376c-3p. Dissociation curve for miRNA rno-miR-377-3p. Dissociation curve for miRNA rno-miR-425-5p. Dissociation curve for miRNA rno-miR-3584-5p. Dissociation curve for U6 (control reference). (ZIP 678 kb)

Additional file 5: **Figure S1.** Gene function classification (GO). (PDF 1761 kb)

Additional file 6: **Table S4.** Results of KEGG pathway analysis. (XLSX 11 kb)

## Abbreviations

DAVID: Database for annotation visualization and integrated discovery
GO: Gene ontology
HSR: Highly sensitive and reproducible
JP2: junctophilin-2
KEGG: Kyoto encyclopedia of genes and genomes
qRT-PCR: Quantitative real-time polymerase chain reaction
RMA: Robust multi-chip analysis
SERCA: Sarco endoplasmic reticulum Ca^2+^-ATPase
VGCC: Voltage gated calcium channels

## References

[1] Kaushal RP, Vatal A, Pathak R (2015). Effect of etomidate and propofol induction on hemodynamic and endocrine response in patients undergoing coronary artery bypass grafting/mitral valve and aortic valve replacement surgery on cardiopulmonary bypass. Ann Card Anaesth.

[2] Masoudifar M, Beheshtian E (2013). Comparison of cardiovascular response to laryngoscopy and tracheal intubation after induction of anesthesia by Propofol and etomidate. Journal of research in medical sciences: the official journal of Isfahan University of Medical. Sciences.

[3] Moller Petrun A, Kamenik M (2013). Bispectral index-guided induction of general anaesthesia in patients undergoing major abdominal surgery using propofol or etomidate: a double-blind, randomized, clinical trial. Br J Anaesth.

[4] Passot S, Servin F, Pascal J, Charret F, Auboyer C, Molliex S (2005). A comparison of target- and manually controlled infusion propofol and etomidate/desflurane anesthesia in elderly patients undergoing hip fracture surgery. Anesth Analg.

[5] Ebert TJ, Muzi M, Berens R, Goff D, Kampine JP (1992). Sympathetic responses to induction of anesthesia in humans with propofol or etomidate. Anesthesiology.

[6] Gelissen HP, Epema AH, Henning RH, Krijnen HJ, Hennis PJ, den Hertog A (1996). Inotropic effects of propofol, thiopental, midazolam, etomidate, and ketamine on isolated human atrial muscle. Anesthesiology.

[7] Yoon SH (2012). Concerns of the anesthesiologist: anesthetic induction in severe sepsis or septic shock patients. Korean J Anesthesiol.

[8] Buljubasic N, Marijic J, Berczi V, Supan DF, Kampine JP, Bosnjak ZJ (1996). Differential effects of etomidate, propofol, and midazolam on calcium and potassium channel currents in canine myocardial cells. Anesthesiology.

[9] Lam CF, Chang PJ, Chen YA, Yeh CY, Tsai YC (2010). Inhibition of ATP-sensitive potassium channels attenuates propofol-induced vasorelaxation. Crit Care Resusc.

[10] Yang M, Ding X, Murray PA (2008). Differential effects of intravenous anesthetics on capacitative calcium entry in human pulmonary artery smooth muscle cells. Am J Physiol Lung Cell Mol Physiol.

[11] Buchan JR, Parker R (2007). Molecular biology. The two faces of miRNA. Science.

[12] Djuranovic S, Nahvi A, Green R (2012). miRNA-mediated gene silencing by translational repression followed by mRNA deadenylation and decay. Science.

[13] Yao Y, Du J, Cao X, Wang Y, Huang Y, Hu S, Zheng Z (2014). Plasma levels of microRNA-499 provide an early indication of perioperative myocardial infarction in coronary artery bypass graft patients. PLoS One.

[14] Montgomery RL, Hullinger TG, Semus HM, Dickinson BA, Seto AG, Lynch JM, Stack C, Latimer PA, Olson EN, van Rooij E (2011). Therapeutic inhibition of miR-208a improves cardiac function and survival during heart failure. Circulation.

[15] Li H, Zhang X, Wang F, Zhou L, Yin Z, Fan J, Nie X, Wang P, Fu XD, Chen C (2016). MicroRNA-21 lowers blood pressure in spontaneous hypertensive rats by upregulating mitochondrial translation. Circulation.

[16] Rothman AM, Arnold ND, Pickworth JA, Iremonger J, Ciuclan L, Allen RM, Guth-Gundel S, Southwood M, Morrell NW, Thomas M (2016). MicroRNA-140-5p and SMURF1 regulate pulmonary arterial hypertension. J Clin Invest.

[17] Vegter EL, van der Meer P, de Windt LJ, Pinto YM, Voors AA (2016). MicroRNAs in heart failure: from biomarker to target for therapy. Eur J Heart Fail.

[18] Wahlquist C, Jeong D, Rojas-Munoz A, Kho C, Lee A, Mitsuyama S, van Mil A, Park WJ, Sluijter JP, Doevendans PA (2014). Inhibition of miR-25 improves cardiac contractility in the failing heart. Nature.

[19] Li X, Wang B, Cui H, Du Y, Song Y, Yang L, Zhang Q, Sun F, Luo D, Xu C (2014). Let-7e replacement yields potent anti-arrhythmic efficacy via targeting beta 1-adrenergic receptor in rat heart. J Cell Mol Med.

[20] Kuster DW, Mulders J, Ten Cate FJ, Michels M, Dos Remedios CG, da Costa Martins PA, van der Velden J, Oudejans CB (2013). MicroRNA transcriptome profiling in cardiac tissue of hypertrophic cardiomyopathy patients with MYBPC3 mutations. J Mol Cell Cardiol.

[21] Sucharov C, Bristow MR, Port JD (2008). miRNA expression in the failing human heart: functional correlates. J Mol Cell Cardiol.

[22] Guo L, Qiu Z, Wei L, Yu X, Gao X, Jiang S, Tian H, Jiang C, Zhu D (2012). The microRNA-328 regulates hypoxic pulmonary hypertension by targeting at insulin growth factor 1 receptor and L-type calcium channel-alpha1C. Hypertension.

[23] Lu Y, Zhang Y, Wang N, Pan Z, Gao X, Zhang F, Zhang Y, Shan H, Luo X, Bai Y (2010). MicroRNA-328 contributes to adverse electrical remodeling in atrial fibrillation. Circulation.

[24] Irizarry RA, Hobbs B, Collin F, Beazer-Barclay YD, Antonellis KJ, Scherf U, Speed TP (2003). Exploration, normalization, and summaries of high density oligonucleotide array probe level data. Biostatistics.

[25] Saeed AI, Bhagabati NK, Braisted JC, Liang W, Sharov V, Howe EA, Li J, Thiagarajan M, White JA, Quackenbush J (2006). TM4 microarray software suite. Methods Enzymol.

[26] Zhang Q, Xiao X, Zheng J, Li M, Yu M, Ping F, Wang Z, Qi C, Wang T, Wang X (2017). Maternal chromium restriction modulates miRNA profiles related to lipid metabolism disorder in mice offspring. Exp Biol Med (Maywood).

[27] Chou CH, Chang NW, Shrestha S, Hsu SD, Lin YL, Lee WH, Yang CD, Hong HC, Wei TY, Tu SJ (2016). miRTarBase 2016: updates to the experimentally validated miRNA-target interactions database. Nucleic Acids Res.

[28] Kanehisa M, Furumichi M, Tanabe M, Sato Y, Morishima K (2017). KEGG: new perspectives on genomes, pathways, diseases and drugs. Nucleic Acids Res.

[29] Dennis G, Sherman BT, Hosack DA, Yang J, Gao W, Lane HC, Lempicki RA (2003). DAVID: database for annotation, visualization, and integrated discovery. Genome Biol.

[30] Martinez-Fernandez A (2014). MicroRNA therapy for the failing heart. Circ Cardiovasc Genet.

[31] Xu M, Wu HD, Li RC, Zhang HB, Wang M, Tao J, Feng XH, Guo YB, Li SF, Lai ST (2012). Mir-24 regulates junctophilin-2 expression in cardiomyocytes. Circ Res.

[32] Li RC, Tao J, Guo YB, Wu HD, Liu RF, Bai Y, Lv ZZ, Luo GZ, Li LL, Wang M (2013). In vivo suppression of microRNA-24 prevents the transition toward decompensated hypertrophy in aortic-constricted mice. Circ Res.

[33] Magenta A, Dellambra E, Ciarapica R, Capogrossi MC (2016). Oxidative stress, microRNAs and cytosolic calcium homeostasis. Cell Calcium.

[34] Boettger T, Braun T (2012). A new level of complexity: the role of microRNAs in cardiovascular development. Circ Res.

