# Supplementary material for: Effects of propofol and etomidate anesthesia on cardiovascular miRNA expression: the different profiles?
Source: BMC Anesthesiol. 2018 Oct 24;18:149. doi: 10.1186/s12871-018-0610-9 (PMC6199805; doi:10.1186/s12871-018-0610-9)
Supplement: Supplementary file 1 — Table S1. Physiological Data for the Propofol and Etomidate Anesthesia Groups. (DOCX 18 kb) [file 12871_2018_610_MOESM1_ESM.docx]

Table S1. Physiological Data for the Propofol and Etomidate Anesthesia Groups

|  | **Etomidate** | |  |  | **Propofol** | |  |
| --- | --- | --- | --- | --- | --- | --- | --- |
|  | **Baseline** | **After injection** | **3h** |  | **Baseline** | **After injection** | **3h** |
| **Body temperature, °C** | 37.1± 0.3 | 37.0± 0.2 | 37.0± 0.2 |  | 37.1± 0.2 | 37.1± 0.1 | 37.1± 0.1 |
| **SpO_2_, %** | 97.0± 1.0 | 96.7± 1.2 | 96.3± 1.2 |  | 96.7± 0.6 | 97.0± 1.0 | 96.7± 0.6 |
| **Heart rate, beats/min** | 341 ± 8 | 339 ± 5 | 339± 11 |  | 338 ± 6 | 284±7*△ | 334 ± 6^#^ |
|  |  |  |  |  |  |  |  |
| **Mean arterial pressure, mmHg** | 118 ± 3 | 115 ± 3 | 117± 3 |  | 118 ±4 | 85 ± 3*△ | 117± 9^#^ |

| Data represent mean ± SD for three animals in each group.  * In the propofol group, compared to baseline, *p* < 0.0001.  # In the propofol group, compared to time point “after injection”, *p* < 0.0001.  △ At the time point “after injection,” compared to the etomidate group, *p* < 0.0001. |
| --- |
