# Supplementary figures and images for: Effects of propofol and etomidate anesthesia on cardiovascular miRNA expression: the different profiles?
Source: BMC Anesthesiol. 2018 Oct 24;18:149. doi: 10.1186/s12871-018-0610-9 (PMC6199805; doi:10.1186/s12871-018-0610-9)

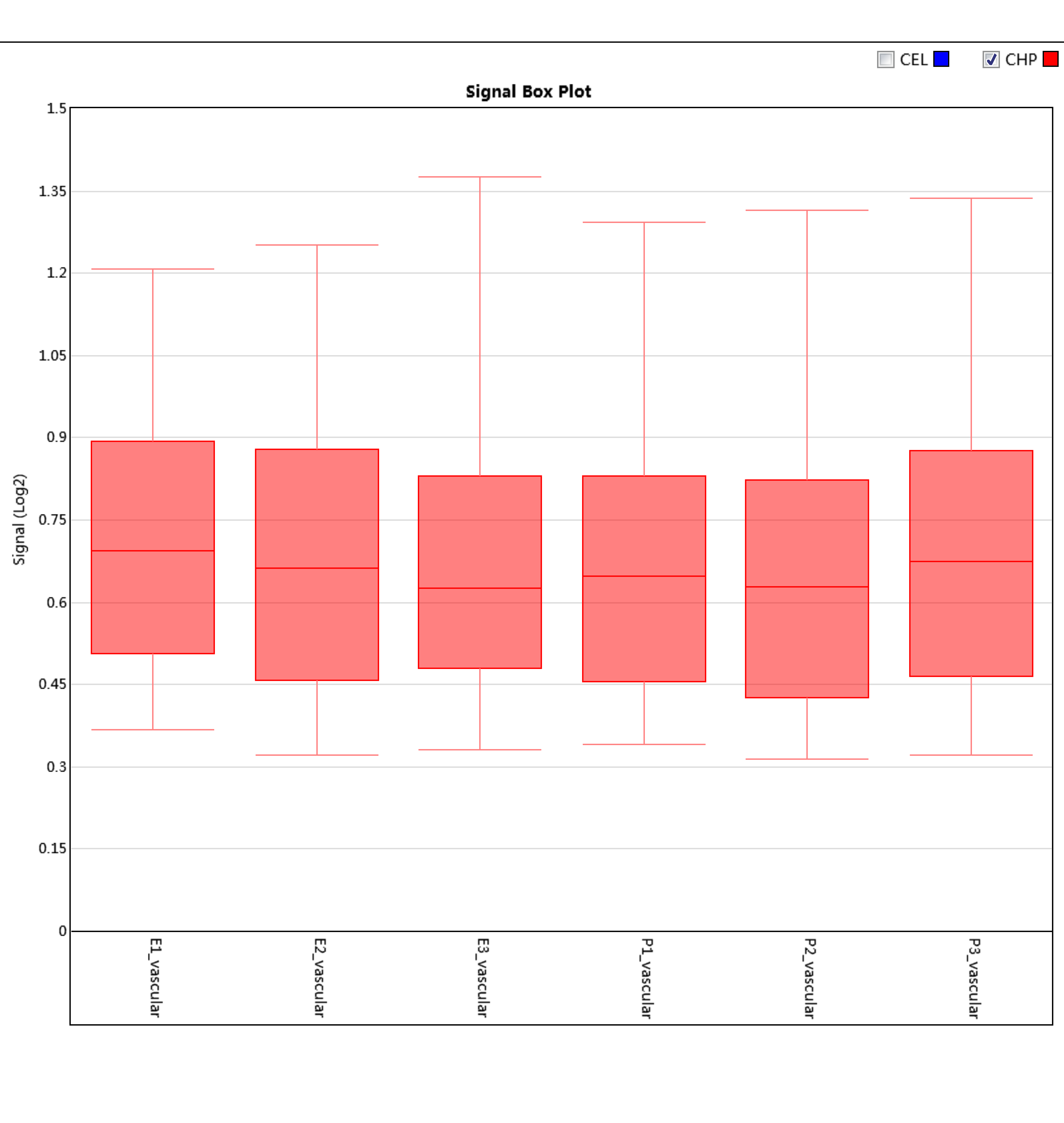

Supplement: Supplementary file 2 — Table S2. Affymetrix original data. Boxplot (vascular). Boxplot (heart). (ZIP 792 kb) [file 12871_2018_610_MOESM2_ESM.zip › Additional file 2-boxplot_vascularR2.png]

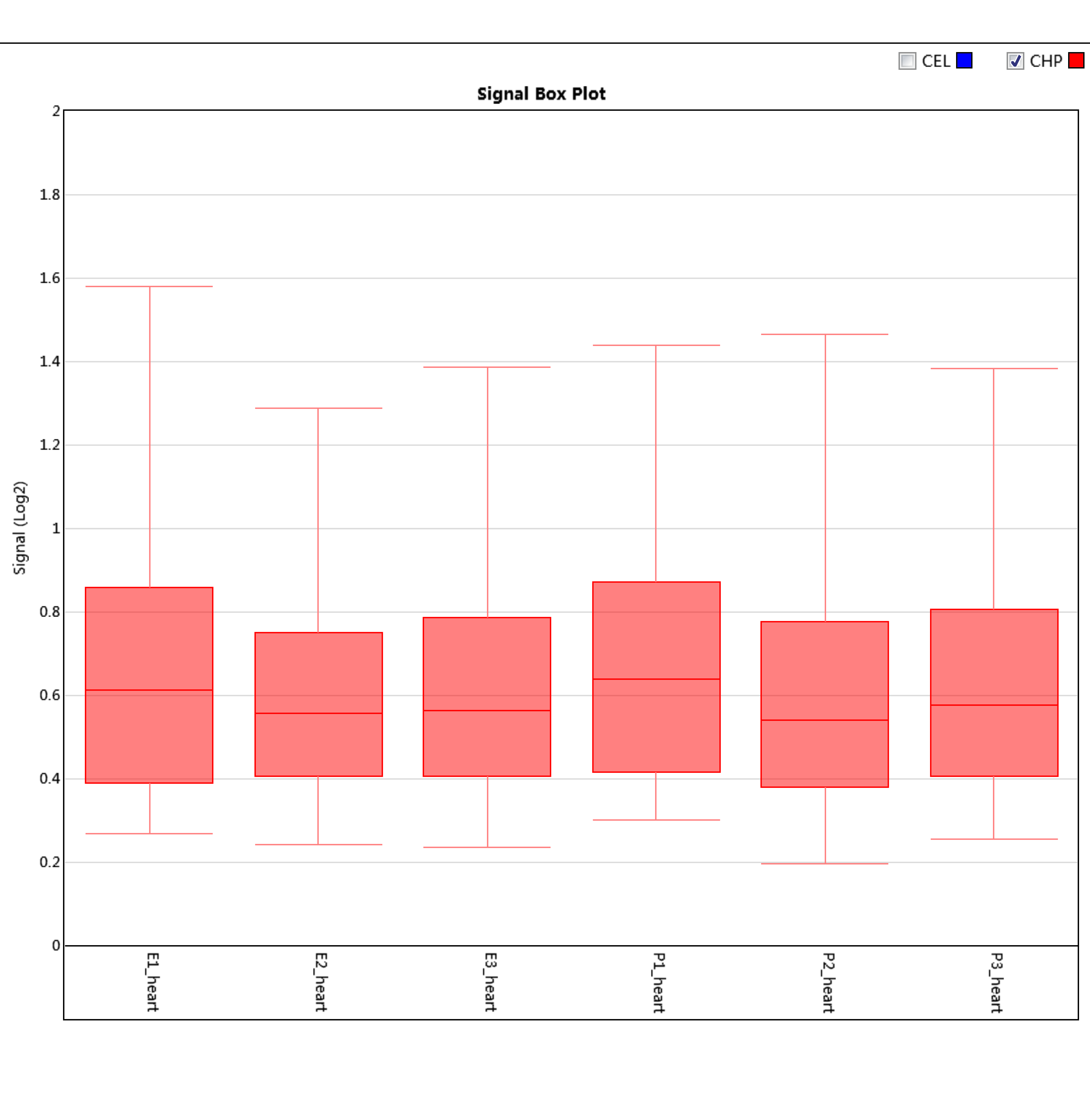

Supplement: Supplementary file 2 — Table S2. Affymetrix original data. Boxplot (vascular). Boxplot (heart). (ZIP 792 kb) [file 12871_2018_610_MOESM2_ESM.zip › Addtional file 2-heart_boxplotR2.png]

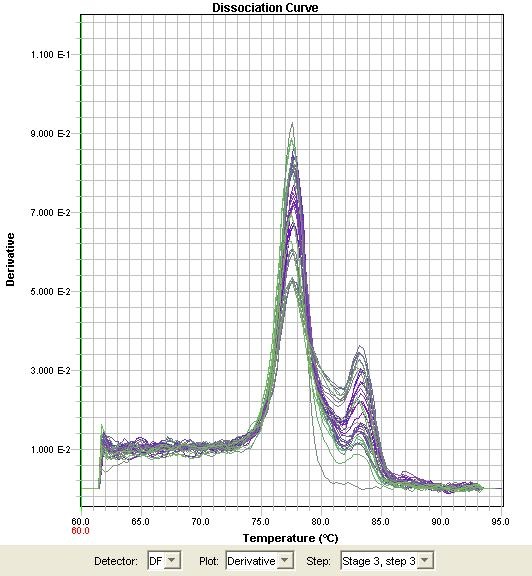

Supplement: Supplementary file 4 — Dissociation curve for miRNA rno-miR-24-3p. Dissociation curve for miRNA rno-mir-93-5p. Dissociation curve for miRNA rno-miR-103-3p. Dissociation curve for miRNA rno-miR-107-3p. Dissociation curve for miRNA rno-mir-129-5p. Dissociation curve for miRNA rno-miR-133-3p. Dissociation curve for miRNA rno-miR-140-3p. Dissociation curve for miRNA rno-miR-320-3p. Dissociation curve for miRNA rno-miR-376c-3p. Dissociation curve for miRNA rno-miR-377-3p. Dissociation curve for miRNA rno-miR-425-5p. Dissociation curve for miRNA rno-miR-3584-5p. Dissociation curve for U6 (control reference). (ZIP 678 kb) [file 12871_2018_610_MOESM4_ESM.zip › rno-miR-103-3pR2.jpg]

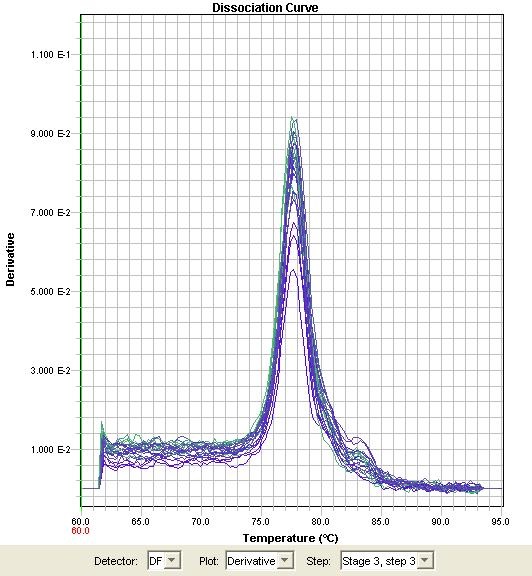

Supplement: Supplementary file 4 — Dissociation curve for miRNA rno-miR-24-3p. Dissociation curve for miRNA rno-mir-93-5p. Dissociation curve for miRNA rno-miR-103-3p. Dissociation curve for miRNA rno-miR-107-3p. Dissociation curve for miRNA rno-mir-129-5p. Dissociation curve for miRNA rno-miR-133-3p. Dissociation curve for miRNA rno-miR-140-3p. Dissociation curve for miRNA rno-miR-320-3p. Dissociation curve for miRNA rno-miR-376c-3p. Dissociation curve for miRNA rno-miR-377-3p. Dissociation curve for miRNA rno-miR-425-5p. Dissociation curve for miRNA rno-miR-3584-5p. Dissociation curve for U6 (control reference). (ZIP 678 kb) [file 12871_2018_610_MOESM4_ESM.zip › rno-miR-107-3pR2.jpg]

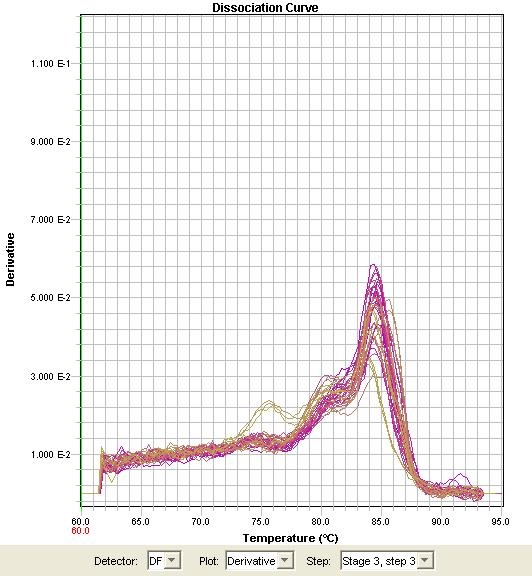

Supplement: Supplementary file 4 — Dissociation curve for miRNA rno-miR-24-3p. Dissociation curve for miRNA rno-mir-93-5p. Dissociation curve for miRNA rno-miR-103-3p. Dissociation curve for miRNA rno-miR-107-3p. Dissociation curve for miRNA rno-mir-129-5p. Dissociation curve for miRNA rno-miR-133-3p. Dissociation curve for miRNA rno-miR-140-3p. Dissociation curve for miRNA rno-miR-320-3p. Dissociation curve for miRNA rno-miR-376c-3p. Dissociation curve for miRNA rno-miR-377-3p. Dissociation curve for miRNA rno-miR-425-5p. Dissociation curve for miRNA rno-miR-3584-5p. Dissociation curve for U6 (control reference). (ZIP 678 kb) [file 12871_2018_610_MOESM4_ESM.zip › rno-mir-129-5pR2.jpg]

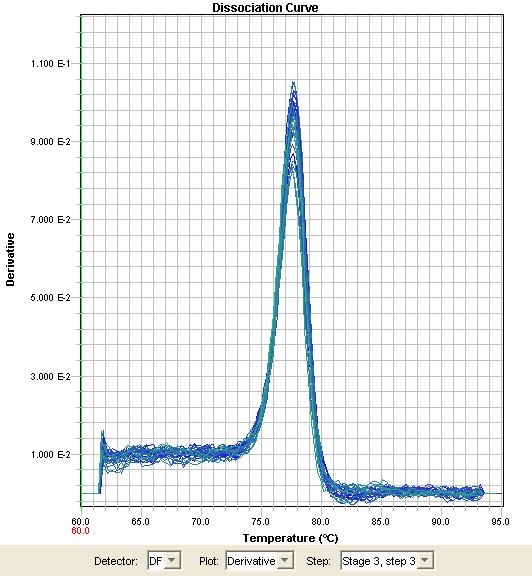

Supplement: Supplementary file 4 — Dissociation curve for miRNA rno-miR-24-3p. Dissociation curve for miRNA rno-mir-93-5p. Dissociation curve for miRNA rno-miR-103-3p. Dissociation curve for miRNA rno-miR-107-3p. Dissociation curve for miRNA rno-mir-129-5p. Dissociation curve for miRNA rno-miR-133-3p. Dissociation curve for miRNA rno-miR-140-3p. Dissociation curve for miRNA rno-miR-320-3p. Dissociation curve for miRNA rno-miR-376c-3p. Dissociation curve for miRNA rno-miR-377-3p. Dissociation curve for miRNA rno-miR-425-5p. Dissociation curve for miRNA rno-miR-3584-5p. Dissociation curve for U6 (control reference). (ZIP 678 kb) [file 12871_2018_610_MOESM4_ESM.zip › rno-miR-133-3pR2.jpg]

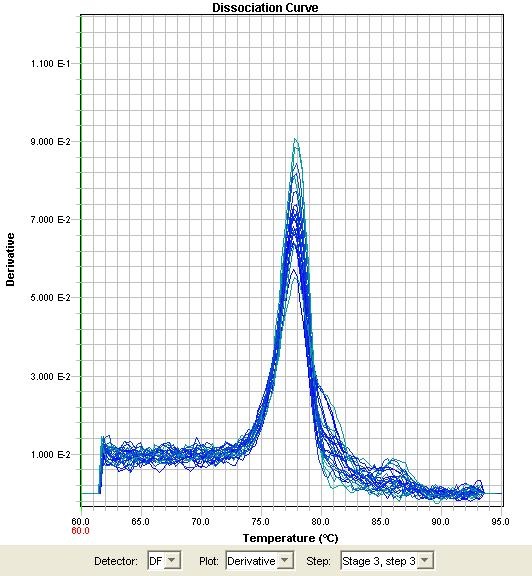

Supplement: Supplementary file 4 — Dissociation curve for miRNA rno-miR-24-3p. Dissociation curve for miRNA rno-mir-93-5p. Dissociation curve for miRNA rno-miR-103-3p. Dissociation curve for miRNA rno-miR-107-3p. Dissociation curve for miRNA rno-mir-129-5p. Dissociation curve for miRNA rno-miR-133-3p. Dissociation curve for miRNA rno-miR-140-3p. Dissociation curve for miRNA rno-miR-320-3p. Dissociation curve for miRNA rno-miR-376c-3p. Dissociation curve for miRNA rno-miR-377-3p. Dissociation curve for miRNA rno-miR-425-5p. Dissociation curve for miRNA rno-miR-3584-5p. Dissociation curve for U6 (control reference). (ZIP 678 kb) [file 12871_2018_610_MOESM4_ESM.zip › rno-miR-140-3pR2.jpg]

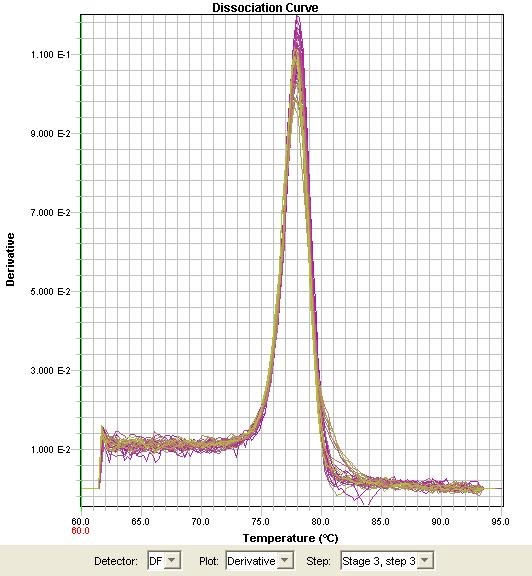

Supplement: Supplementary file 4 — Dissociation curve for miRNA rno-miR-24-3p. Dissociation curve for miRNA rno-mir-93-5p. Dissociation curve for miRNA rno-miR-103-3p. Dissociation curve for miRNA rno-miR-107-3p. Dissociation curve for miRNA rno-mir-129-5p. Dissociation curve for miRNA rno-miR-133-3p. Dissociation curve for miRNA rno-miR-140-3p. Dissociation curve for miRNA rno-miR-320-3p. Dissociation curve for miRNA rno-miR-376c-3p. Dissociation curve for miRNA rno-miR-377-3p. Dissociation curve for miRNA rno-miR-425-5p. Dissociation curve for miRNA rno-miR-3584-5p. Dissociation curve for U6 (control reference). (ZIP 678 kb) [file 12871_2018_610_MOESM4_ESM.zip › rno-miR-24-3pR2.jpg]

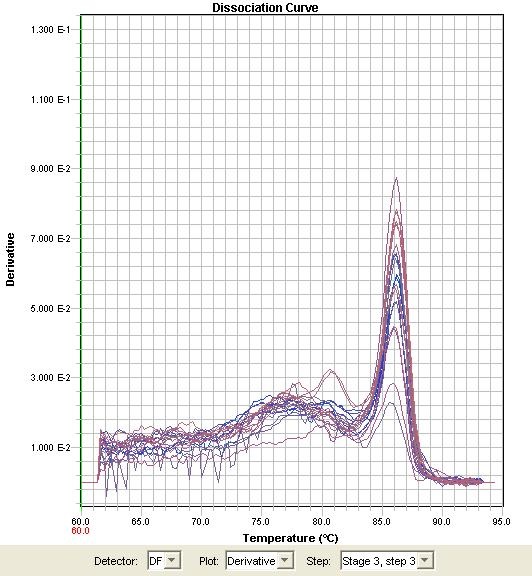

Supplement: Supplementary file 4 — Dissociation curve for miRNA rno-miR-24-3p. Dissociation curve for miRNA rno-mir-93-5p. Dissociation curve for miRNA rno-miR-103-3p. Dissociation curve for miRNA rno-miR-107-3p. Dissociation curve for miRNA rno-mir-129-5p. Dissociation curve for miRNA rno-miR-133-3p. Dissociation curve for miRNA rno-miR-140-3p. Dissociation curve for miRNA rno-miR-320-3p. Dissociation curve for miRNA rno-miR-376c-3p. Dissociation curve for miRNA rno-miR-377-3p. Dissociation curve for miRNA rno-miR-425-5p. Dissociation curve for miRNA rno-miR-3584-5p. Dissociation curve for U6 (control reference). (ZIP 678 kb) [file 12871_2018_610_MOESM4_ESM.zip › rno-miR-320-3pR2.jpg]

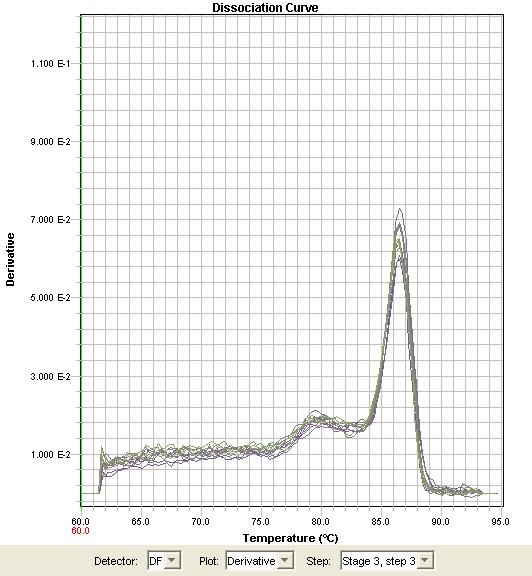

Supplement: Supplementary file 4 — Dissociation curve for miRNA rno-miR-24-3p. Dissociation curve for miRNA rno-mir-93-5p. Dissociation curve for miRNA rno-miR-103-3p. Dissociation curve for miRNA rno-miR-107-3p. Dissociation curve for miRNA rno-mir-129-5p. Dissociation curve for miRNA rno-miR-133-3p. Dissociation curve for miRNA rno-miR-140-3p. Dissociation curve for miRNA rno-miR-320-3p. Dissociation curve for miRNA rno-miR-376c-3p. Dissociation curve for miRNA rno-miR-377-3p. Dissociation curve for miRNA rno-miR-425-5p. Dissociation curve for miRNA rno-miR-3584-5p. Dissociation curve for U6 (control reference). (ZIP 678 kb) [file 12871_2018_610_MOESM4_ESM.zip › rno-miR-3584-5pR2.jpg]

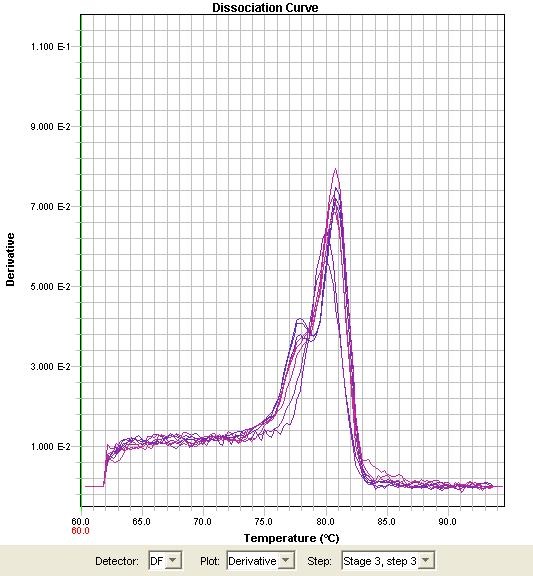

Supplement: Supplementary file 4 — Dissociation curve for miRNA rno-miR-24-3p. Dissociation curve for miRNA rno-mir-93-5p. Dissociation curve for miRNA rno-miR-103-3p. Dissociation curve for miRNA rno-miR-107-3p. Dissociation curve for miRNA rno-mir-129-5p. Dissociation curve for miRNA rno-miR-133-3p. Dissociation curve for miRNA rno-miR-140-3p. Dissociation curve for miRNA rno-miR-320-3p. Dissociation curve for miRNA rno-miR-376c-3p. Dissociation curve for miRNA rno-miR-377-3p. Dissociation curve for miRNA rno-miR-425-5p. Dissociation curve for miRNA rno-miR-3584-5p. Dissociation curve for U6 (control reference). (ZIP 678 kb) [file 12871_2018_610_MOESM4_ESM.zip › rno-miR-376c-3pR2.jpg]

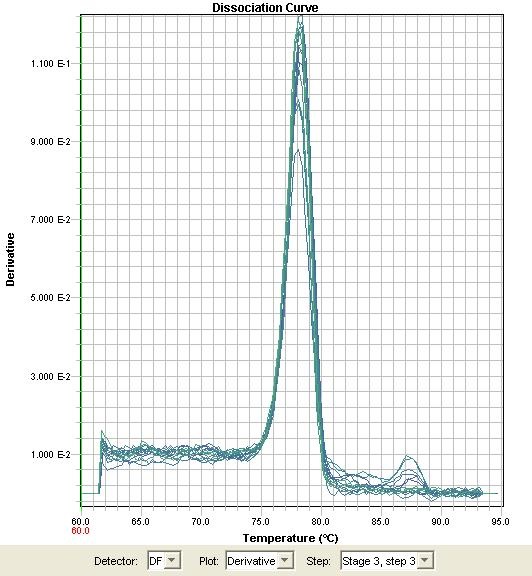

Supplement: Supplementary file 4 — Dissociation curve for miRNA rno-miR-24-3p. Dissociation curve for miRNA rno-mir-93-5p. Dissociation curve for miRNA rno-miR-103-3p. Dissociation curve for miRNA rno-miR-107-3p. Dissociation curve for miRNA rno-mir-129-5p. Dissociation curve for miRNA rno-miR-133-3p. Dissociation curve for miRNA rno-miR-140-3p. Dissociation curve for miRNA rno-miR-320-3p. Dissociation curve for miRNA rno-miR-376c-3p. Dissociation curve for miRNA rno-miR-377-3p. Dissociation curve for miRNA rno-miR-425-5p. Dissociation curve for miRNA rno-miR-3584-5p. Dissociation curve for U6 (control reference). (ZIP 678 kb) [file 12871_2018_610_MOESM4_ESM.zip › rno-miR-377-3pR2.jpg]

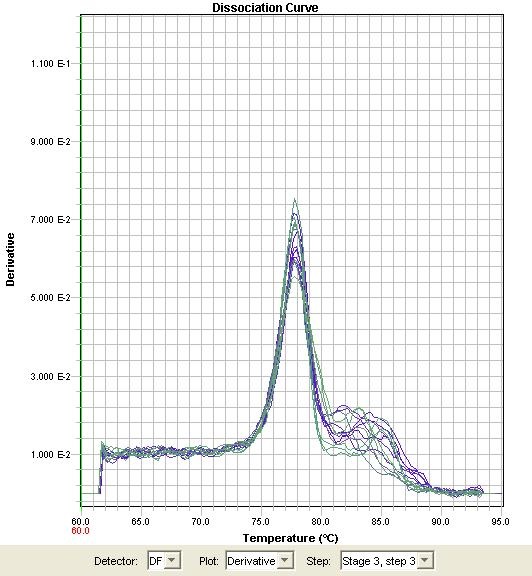

Supplement: Supplementary file 4 — Dissociation curve for miRNA rno-miR-24-3p. Dissociation curve for miRNA rno-mir-93-5p. Dissociation curve for miRNA rno-miR-103-3p. Dissociation curve for miRNA rno-miR-107-3p. Dissociation curve for miRNA rno-mir-129-5p. Dissociation curve for miRNA rno-miR-133-3p. Dissociation curve for miRNA rno-miR-140-3p. Dissociation curve for miRNA rno-miR-320-3p. Dissociation curve for miRNA rno-miR-376c-3p. Dissociation curve for miRNA rno-miR-377-3p. Dissociation curve for miRNA rno-miR-425-5p. Dissociation curve for miRNA rno-miR-3584-5p. Dissociation curve for U6 (control reference). (ZIP 678 kb) [file 12871_2018_610_MOESM4_ESM.zip › rno-miR-425-5pR2.jpg]

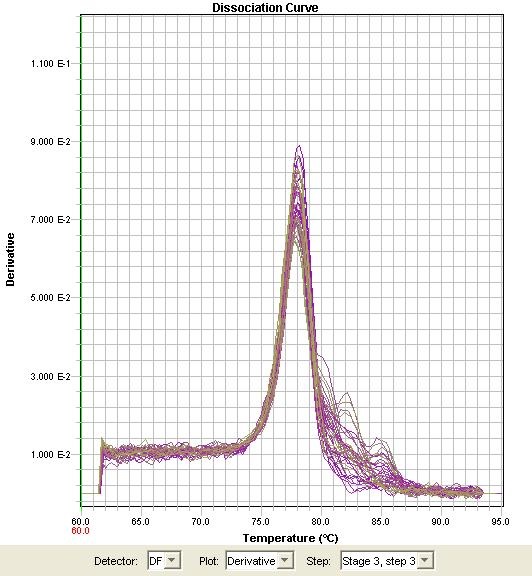

Supplement: Supplementary file 4 — Dissociation curve for miRNA rno-miR-24-3p. Dissociation curve for miRNA rno-mir-93-5p. Dissociation curve for miRNA rno-miR-103-3p. Dissociation curve for miRNA rno-miR-107-3p. Dissociation curve for miRNA rno-mir-129-5p. Dissociation curve for miRNA rno-miR-133-3p. Dissociation curve for miRNA rno-miR-140-3p. Dissociation curve for miRNA rno-miR-320-3p. Dissociation curve for miRNA rno-miR-376c-3p. Dissociation curve for miRNA rno-miR-377-3p. Dissociation curve for miRNA rno-miR-425-5p. Dissociation curve for miRNA rno-miR-3584-5p. Dissociation curve for U6 (control reference). (ZIP 678 kb) [file 12871_2018_610_MOESM4_ESM.zip › rno-mir-93-5pR2.jpg]

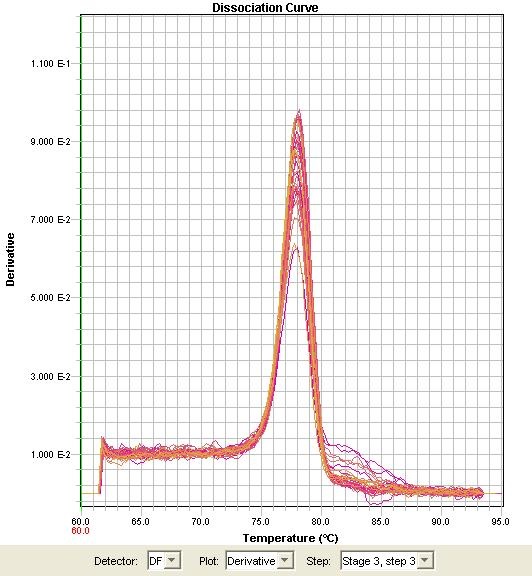

Supplement: Supplementary file 4 — Dissociation curve for miRNA rno-miR-24-3p. Dissociation curve for miRNA rno-mir-93-5p. Dissociation curve for miRNA rno-miR-103-3p. Dissociation curve for miRNA rno-miR-107-3p. Dissociation curve for miRNA rno-mir-129-5p. Dissociation curve for miRNA rno-miR-133-3p. Dissociation curve for miRNA rno-miR-140-3p. Dissociation curve for miRNA rno-miR-320-3p. Dissociation curve for miRNA rno-miR-376c-3p. Dissociation curve for miRNA rno-miR-377-3p. Dissociation curve for miRNA rno-miR-425-5p. Dissociation curve for miRNA rno-miR-3584-5p. Dissociation curve for U6 (control reference). (ZIP 678 kb) [file 12871_2018_610_MOESM4_ESM.zip › U6R2.jpg]

# Gene Function Classification (GO)

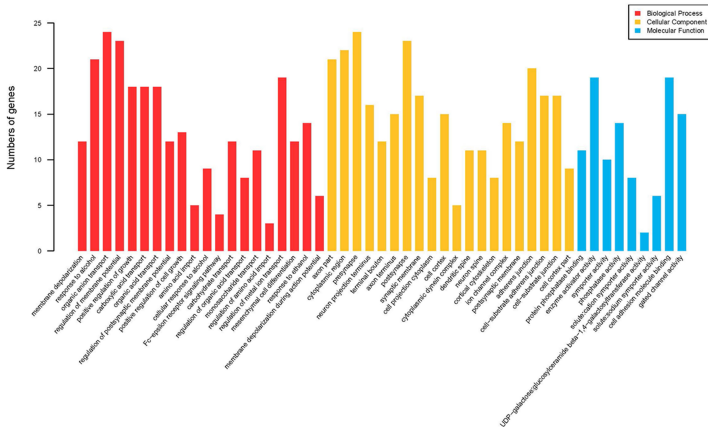

Supplement: Supplementary file 5 — Figure S1. Gene function classification (GO). (PDF 1761 kb) [file 12871_2018_610_MOESM5_ESM.pdf]
